# The Effect of Nursing Internships on the Effectiveness of Implementing Information Technology Teaching

**DOI:** 10.3389/fpubh.2022.893199

**Published:** 2022-05-13

**Authors:** Li-Ping Tseng, Tung-Hsu Hou, Li-Ping Huang, Yang-Kun Ou

**Affiliations:** ^1^Department of Management Center, Sisters of Our Lady of China Catholic Medical Foundation, St. Martin De Porres Hospital, Chiayi, Taiwan; ^2^Department of Industrial Engineering and Management, National Yunlin University of Science and Technology, Douliu, Taiwan; ^3^Department of Nursing, Chung-Jen Junior College of Nursing, Health Sciences and Management, Chiayi, Taiwan; ^4^Department of Creative Product Design, Southern Taiwan University of Science and Technology, Tainan, Taiwan

**Keywords:** patient simulation, awareness, nursing education, information technology, OSCE

## Abstract

In nursing education, the diversity of clinical scenarios is complex and dynamic, and it is a challenge for nursing students to learn this clinical knowledge in conventional teaching mechanisms. However, integrating information technology into teaching can promote diversity of learning environment. This study aimed to investigate the effectiveness of teaching mechanisms which combined clinical simulation scenario and Information Technology Integrated Instruction. This study applied innovative experimental teaching in medical–surgical and critical care nursing courses for nursing students in their fourth or fifth year at a five-year junior college. Instructional systems design was combined with clinical simulation scenarios and technology to develop multidimensional teaching strategies. Fifth-year students' overall OSCE mean score was considerably higher than that of fourth-year students; specifically, their scores on basic life support and care for subdural hemorrhage varied substantially. Compared with fourth-year students, fifth-year students performed considerably greater on situational awareness. The results of the present study can be used to develop nursing core competencies and improve the clinical care competency of nursing personnel.

## Introduction

Objective structured clinical examination (OSCE) is essentially a method for assessing learning outcomes by measuring clinical nursing ability and competence in clinical practice (1, 2). To conduct OSCE, a series of assessment stations is established, various health problems are simulated, and students' behavior and interaction with standardized patients are video recorded. Watson et al. (2) defined OSCE as an examination in which “students demonstrate their competence under a variety of simulated conditions.” OSCE is effective for presenting learning outcomes and assessing students' clinical performance after graduation (1). Situational awareness (SA) describes individuals' perception, understanding, and subsequent predictions of what will happen in the surrounding environment. Specifically, people with high SA are aware of their surroundings and use that information to devise necessary plans or make decisions (3, 4). Projection is the highest level of SA and is achieved through personal understanding and the interpretation of perceptual clues as well as the expectation of future results (5, 6). Nursing care is a dynamic process, and Tower et al. (7) identified clinical decision-making as a paramount skill for nurses when caring for patients. High SA helps nursing personnel make appropriate clinical decisions vital to patient care outcomes and safety. SA training for nursing personnel should be improved to help them achieve the highest level of SA and provide high-quality nursing care. The rapid development of information technology and prevalence of the internet have rendered information skills essential to student competitiveness (8). Innovative teaching methods such as online education, massive open online courses (MOOCs), and small private online courses (SPOCs) have been developed and employ various interactive educational software programs, high-fidelity equipment, teaching platforms, and sharing platforms that differ considerably from conventional teaching methods (9). The application of information integration has also changed how researchers examine learning methods (10, 11). The integration of technology into nursing education for the cultivation of professional nursing skills warrants further discussion.

This study applied innovative experimental teaching in medical–surgical and critical care nursing courses for nursing students in their fourth or fifth year at a 5-year junior college. Instructional systems design was combined with clinical simulation scenarios and technology to develop multidimensional teaching strategies. The flipped classroom approach and lesson plans based on clinical scenario simulations were applied to establish a reliable and valid OSCE scale for assessing nursing students' learning effectiveness, SA, and satisfaction with the proposed teaching methods. The findings may serve as a reference and highlight valuable implications for the development and application of future nursing education programs. The objectives of this study are as follows:

(1) To establish clinical simulation scenario lesson plans for medical and surgical nursing and integrate it with an OSCE, and then to verify the reliability and validity of the OSCE Checklist.
(2) Explore the difference between fourth- and fifth-year students in terms of OSCE scores following their participation in innovative teaching courses.
(3) Explore the difference between fourth- and fifth-year students in SA performance following their participation in innovative teaching courses.
(4) Explore fourth- and fifth-year students' satisfaction with the innovative teaching courses.

## Materials and Methods

### Participants

Approval was obtained from the Institutional Review Board of St. Martin De Porres Hospital (No. 18B-12). Purposive sampling was conducted to select fourth- and fifth-year nursing students from a 5-year health care and management junior college in central Taiwan. Initially, 216 fourth-year nursing students and 74 fifth-year nursing students were recruited prior to the first semester of 2019. Researchers visited each class to explain the research plan, including the research objectives, research process, the rights of the participants, the implementation of the innovative courses, the teaching methods based on clinical scenario simulation and technology integration, administration of revised OSCE, and the required tasks. The students recruited for the study provided informed consent to human research after sufficient time for consideration. In total, 126 students constituted of 61 fourth-year and 65 fifth-year students participated in the study. Data was collected from March 1st, 2019 through to October 30th, 2020.

### Research Tools

#### Medical and Surgical Nursing Courses

In the national examination, medical and surgical nursing is a core competency course and a major subject. This course cultivates students' professional and practical capacity in medical and surgical nursing—applying basic biomedical and scientific knowledge and nursing expertise they have learned; assessing and analyzing patients' physical, mental, spiritual, and sociocultural responses; suitably providing nursing measures; and accurately implementing and applying related techniques to clinical practices. This research implements innovative teaching methods, with teaching strategies including lectures, flipped classrooms, group discussions, information-technology based strategies, simulated scenarios and OSCE to cover the following eight topics of nursing care: body fluid and electrolyte imbalance, respiratory system, cardiovascular system, basic and advanced life support, shock, sepsis, and multiple organ failure, endocrine disorders, nervous system and urinary system.

#### Course Development and Design of Clinical Simulation Scenario Lesson Plans

There were two stages to develop course and design clinical simulation scenario lesson plans in the present study.

Stage 1: The medical–surgical and critical nursing courses integration.

The course development team, which constituted by nursing instructors, clinical nursing experts, and a clinical physician, convened several teaching discussion meetings to integrate teaching and learning mechanisms aimed at fourth-year students of a 5-year nursing program. They also incorporated scenario simulation with ITII (Information Technology Integrated Instruction) as the teaching strategy and conducted flipped classroom concepts and a revised OSCE to establish a critical nursing course. Traditional OSCE is usually conducted with rotations among multiple stations, while the three OSCE subjects tested in this study among 126 students, due to constraints in time, space and examiner availability were conducted by the examiners scoring the video recordings of students performing the tasks in the skills lab. The revised OSCE was more effective and fulfills its purpose than the traditional.

Stage 2: Scenario simulation teaching and clinical scenario teaching templates with the ADDIE model development.

(1) Scenario simulation teaching and clinical scenario teaching templates development

The present study introduced scenario simulation teaching into the lesson plans by referencing the Human Patient Simulation Scenario Development Patient Case Template (HPSSDPCT), the Template of Events for Applied and Critical Healthcare Simulation (TEACH Sim) (12) and the ADDIE teaching design model which included analysis, design, development, implementation, and evaluation. The learning priorities, important events, and target responses were set by KSA (knowledge, skills, and attitude) which involved cognitive, psychomotor and affective domains.

(2) The clinical scenario simulation lesson plans design

The five topics was developed by course development team, included percutaneous transluminal coronary angioplasty (PTCA) and stent placement care for patients with acute myocardial infarction (AMI), basic life support (BLS) and operation of automated external defibrillator (AED), subdural hemorrhage (SDH) care, applications of the advanced Apollo Simulator in care for patients with septic shock, and acute respiratory distress syndrome (ARDS) care. They select the these five topics based on the importance and feasibility of the major teaching contents, and integrate the knowledge of other teaching contents into them as much as possible. Then, the final version was formed by conducting two pilot tests. Depending on the topic scenario, each plan was taught for 30–90 min.

### OSCE Checklist Development

OSCE scores were used to evaluate student performance in this study and the test of face validity, content validity, and criterion validity were conduct after the first draft of the OSCE checklists.

(1) The First Draft of the Checklists

Because septic shock care and ARDS care require the use of advanced simulators and are therefore limited by equipment and this study's OSCE feasibility, these two scenarios were mainly taught and excluded from the OSCEs. In the end, the PTCA care for patients with AMI, BLS and AED operation, and SDH care were included in OSCE checklists. Checklist items were based on the actions in the process that students must complete; each checklist item was graded in 0 to 2 points (2 = fully completed, 1 = partially completed, 0 = not completed).

(2) Expert Content Validity Tests

The seventeen clinical experts and three fifth-year nursing students were invited to review the first draft of the three clinical scenario checklists and this study used the content validity index (CVI) as the measurement, each item of the evaluation survey was assigned 1–4 points on a 4-point Likert scale, where 1 = very appropriate, 2 = appropriate, 3 = inappropriate, and 4 = very inappropriate. The items were assessed based on expert appraisal, and the statistical method was based on the number of appropriate/very appropriate items divided by the total number of items; if the result was >0.80, the checklist was indicated to have a favorable validity index (13). The expert evaluations and opinions were organized into a summary table. Items that received 3 or 4 points were retained; items that received only 1 or 2 points or were determined to be unclear or badly worded were either revised or deleted after the expert opinions were discussed by the course development team. The CVI of PTCA care for patients with AMI was 0.981; 26 items were retained (four items were revised, one item was deleted). The CVI of BLS and AED was 0.987; 25 items were retained (three items were revised). The CVI of SDH care was 0.981; 13 items were retained (one item was revised). The OSCE checklists of all three simulation scenarios were graded based on a full score of 100 points.

(3) Reliability Testing

Four examiners used the Kendall coefficient of concordance to evaluate inter-rater reliability of checklists. For each topic, six students were sampled and the revised OSCE of the clinical scenario was held in the clinical skills center. Four nursing instructors evaluated the students' performances based on the three revised checklists. The Kendall coefficient of concordance values for the three clinical scenarios were 0.937, 0.896, and 0.965, suggesting high consistency; chi-square values were 18.731, 17.793, and 19.307 with *p*-values 0.000, 0.003, and 0.002, all smaller than 0.05. The performance of six students were significantly different, but the consistency in OSCE checklist evaluation was high among four instructors, indicating the revised OSCE checklist to be valid.

(4) Determination of SA Levels

Three SA levels were determined by 2 clinical design experts and three human-factors engineering experts and were integrated into OSCE checklists for assessment. Additionally, the present study also refered to Thompson et al. (3) and Tower et al. (7) for the applications of SA levels in medicine: the perception of cues from the setting, including patient information, electronic medical charts and information from other clinicians is important for processing of information and subsequent decision-making for the organization, rationalization and ordering of usuable data to construct an understanding of the present situation in the system to predict one or more possible occurrence of events to formulate appropriate responses and predict the next step. The first level (SA1) is perception level which included basic patient identification, interpretations, and skills operations. The second level (SA2) is comprehension level which included further treatment following observation, decisions and treatments based on SA1. The last level (SA3) is projection level which included proposal of precautions and health education, decisions and treatment based on SA2.

### Information Technology Integrated Instruction Application

According to course progress and clinical scenario topics, seven information technology tools inclueded SPOCs, Zuvio, MOODLE teaching platform, LINE APP, High-fidelity wireless simulaton, Little Anne QCPR and AED trainer, were introduced in this study.

### Satisfaction Survey

The student satisfaction survey was a structured survey modified with permission from a twenty-item survey by Tsai (14) with items relating to simulated clinical scenario, SPOCs and Zuvio rated by a five-point Likert scale to be submitted by nursing students anonymously for quantitative anlalyses.

## Results

In total, 61 fourth-year and 65 fifth-year nursing students were recruited from a 5-year junior college. SPSS Statistics 23.0 was used for data compiling and analysis. Applied statistical methods consisted of descriptive statistics, independent sample *t*-tests, and Pearson's correlation coefficients. This section describes the reliability analysis of the scoring scales for the three OSCE dimensions, the effectiveness of the teaching methods according to clinical scenario simulation and technology integration, and student satisfaction with the innovative teaching methods.

### Reliability Analysis of the Three OSCE Checklists

The consistency of the three OSCE checklists for the AMI, BLS, and SDH lesson plans were verified using Cronbach's α; the results were 0.61 for the AMI checklist, 0.80 for the BLS checklist, and 0.76 for the SDH checklist. The BLS and SDH checklists achieved high reliability.

### Learning Performance of the Clinical Simulation Scenarios and ITII

#### Correlation Between Three Items of OSCE Score and Students' Scores on the Summative Critical Care Nursing Assessment

The correlation between summative evaluations of fourth and fifth year students and the revised OSCE scores, and to utilize the summative scores as performance indices for the OSCE subjects. The showed the results of fourth-year students showed that significant correlation between their summative critical care nursing assessment and revised OSCE score for AMI [*r*(61) = 0.587, *p* = 0.000], BLS [*r*(61) = 0.279, *p* = 0.029], SDH [*r*(61) = 0.471, *p* = 0.000]. The fifth-year students also showed that significant correlation between their summative critical care nursing assessment and revised OSCE score for AMI [*r*(65) = 0.289, *p* = 0.020], BLS [*r*(65) = 0.303, *p* = 0.014], SDH [*r*(65) = 0.389, *p* = 0.001]. Significant positive correlation between three items of OSCE score and scores on the summative critical care nursing assessment was found in the fourth-year and fifth-year students group (Table 1).

**Table 1:** Pearson correlation coefficients between three items of OSCE score and fourth-year and fifth-year students' scores on the summative critical care nursing assessment.

**Variables**	**Fourth-year students (*****n*** **=** **61)**		**Fifth-year students (*****n*** **=** **65)**	
	**Pearson correlation coefficient**	***p*-value (two-tailed)**	**Pearson correlation coefficient**	***p*-value (two-tailed)**
OSCE_AMI	0.587	0.000^**^	0.289	0.020^*^
OSCE_BLS	0.279	0.029^*^	0.303	0.014^*^
OSCE_SDH	0.471	0.000^**^	0.389	0.001^**^

** *p < 0.01*,

* *p < 0.05*.

#### Difference Between Fourth- and Fifth-Year Students in Overall Revised OSCE Scores

The fifth-year students had completed a year-long clinical nursing internship, whereas the fourth-year students were preparing for the internship after the completion of the prior semester. The results of the independent sample *t*-test for the two groups are presented in Table 2. The results revealed that the scores of fourth- and fifth-year students on the summative critical care nursing assessment [*t*(61.65) = −1.22, *p* = 0.225] differed non-significantly. The slightly higher mean score for fifth-year students indicated that internship experience did not affect student performance in critical care nursing. The results showed that two groups of students differed significantly in overall revised OSCE scores [*t*(61.65) = −8.34, *p* < 0.001], and the fifth-year students had a total mean score 33 points higher than the fourth-year students did (Table 2).

**Table 2:** Scores of fourth- and fifth-year students on the summative critical care nursing assessment and OSCE.

**Variables**	**Fourth-year students (*****n*** **=** **61)**		**Fifth-year students (*****n*** **=** **65)**		***t*-value**	**Degree of freedom**	**Significance (two-tailed)**
	**M**	**SD**	**M**	**SD**			
Summative critical care nursing assessment	80.30	5.743	81.88	8.473	−1.219	124	0.225
Overall revised OSCE	230.18	26.890	263.43	17.062	−8.340	124	0.000^**^
OSCE_AMI	84.49	15.802	85.86	8.305	−0.614	124	0.540
OSCE_BLS	85.21	8.456	94.80	6.546	−7.141	124	0.000^**^
OSCE_SDH	60.57	11.566	82.62	10.087	−11.419	124	0.000^**^

** *p < 0.01*.

#### Difference Between Fourth- and Fifth-Year Students in Scores on the Three OSCE Dimensions

The following results were obtained (Table 1): Fourth- and fifth-year students exhibited significant differences in BLS [*t*(61.65) = −7.14, *p* < 0.001] and SDH [*t*(61.65) = −11.42, *p* < 0.001]. Compared with fourth-year students, fifth-year students scored 9.59 and 22.05 points higher for mean BLS and mean SDH, respectively. Despite the non-significant difference in AMI, fifth-year students still had a higher mean score for this dimension than fourth-year students did.

#### SA Differences Between Fourth- and Fifth-Year Students

All participants were assessed for SA1, SA2, and SA3 on the AMI, BLS, and SDH in the OSCE. The following results were obtained (Table 3): The two groups of students exhibited significant differences in AMI SA1 [*t*(61.65) = −3.80, *p* < 0.001]; SA1 [*t*(61.65) = −6.41, *p* < 0.001], SA2 [*t*(61.65) = −4.93, *p* < 0.001], BLS SA3 [*t*(61.65) = −3.92, *p* = 0.001], and SDH SA3 [*t*(61.65) = −18.17, *p* < 0.001]. The fifth-year students were higher scores than those of the fourth-year students, with a considerable 21.29-point difference in SDH SA3.

**Table 3:** Scores of fourth- and fifth-year students on the summative critical care nursing assessment and OSCE.

**Variables**	**Fourth-year students (*****n*** **=** **61)**		**Fifth-year students (*****n*** **=** **65)**		***t*-value**	**Degree of freedom**	**Significance (two-tailed)**
	**M**	**SD**	**M**	**SD**			
AMI_SA1	23.92	4.394	26.14	1.657	−3.797	124	0.000^**^
AMI_SA2	30.98	6.896	29.49	4.272	1.469	124	0.144
AMI_SA3	29.46	7.435	30.88	4.498	−1.304	124	0.195
BLS_SA1	44.52	4.660	49.57	4.176	−6.407	124	0.000^**^
BLS_SA2	22.72	3.984	25.72	2.781	−4.929	124	0.000^**^
BLS_SA3	17.97	2.840	19.51	1.371	−3.915	124	0.000^**^
SDH_SA1	21.66	3.803	22.20	1.897	−1.026	124	0.307
SDH_SA2	25.89	5.404	25.92	5.048	−0.041	124	0.968
SDH_SA3	13.00	6.807	34.29	6.346	−18.172	124	0.000^**^

** *p < 0.01*.

#### SPOC Completion Rate of Fourth- and Fifth-Year Students

In total, 20 SPOCs were established in this study, and students were able to preview lessons through the flipped classroom approach. The shortest and longest SPOCs, which were entitled “Bonus: Virtual Reality for BLS and AMI” and “BLS,” were 7 min and 34 s and 44 min and 25 s long, respectively. Most SPOCs were between 20 and 30 min, with a mean duration of 20 min and 10 s. The SPOC completion rate was calculated by the number of fourth or fifth year students online for the duration of the class divided by the total number of fourth or fifth year students recruited for this study, multiplied by one hundred percent. The difference of SPOC completion rates between fourth- and fifth-year students was non-significant (fourth year: 77.5%; fifth year: 75.8%). For the courses “Respiratory System and Review,” “Bonus: Virtual Reality for BLS and AMI,” and “Medical–Surgical Nursing for Cerebrovascular Diseases,” students had higher completion rate than fourth-year students by 10–30%. For the courses “Endocrine System and Function,” “Physiology of the Nervous System,” “Abdominal Physical Assessment and Common Diseases,” and “Gastrointestinal Bleed Care,” the completion rate for fourth-year students was higher than that for fifth-year students by more than 20%. The SPOC completion rate statistics are presented in Table 4.

**Table 4:** SPOC completion rate statistics.

**Course**	**Duration** **(min:s)**	**Completion rate for fourth-year students (%)**	**Completion rate for fifth-year students (%)**
Basic hemodynamics	08:23	88.73	94.74
Overall revised OSCE	−8.340	124	0.000^**^
Anatomy and physiology of respiratory system and review	14:53	78.87	88.16
Chest x ray reading	18:04	77.46	78.95
Respiratory system treatment and care	18:44	73.24	77.63
EKG (1)	30:29	84.51	81.58
EKG (2)	22:25	74.65	78.95
EKG (3)	18:20	71.83	71.05
Medical–surgical nursing for coronary artery diseases	28:15	76.06	68.42
Medical–surgical nursing for AMI and review	17:35	66.20	61.84
BLS	44:25	70.42	61.84
Bonus: virtual reality for BLS and AMI	07:34	56.34	86.84
Wound care physiology and review	16:53	90.14	82.89
Fracture healing and care	15:45	80.28	84.21
Endocrine system and function	20:07	88.73	71.05
Physiology of the nervous system	27:27	83.10	60.53
Medical–surgical nursing for cerebrovascular diseases	27:37	80.28	89.47
Care for increased ICP	16:26	83.10	90.79
Abdominal physical assessment and common diseases	17:05	69.01	52.63
Gastrointestinal bleed care	11:22	77.46	48.68
Mean	20:10	77.53	75.80

** *p < 0.01*.

### Student Satisfaction With Innovative Teaching Methods

The overall satisfaction scale used in the current study covered three dimensions, namely scenario-based simulation teaching, SPOCs, and the interactive educational software program Zuvio, and comprised 18 items. The overall satisfaction scale yielded a Cronbach's alpha of 0.984, indicating high reliability. The fourth- and fifth-year students varied in terms of items they were satisfied with and in satisfaction with the three dimensions covered by the scale.

The following results were obtained (Table 5): Our results showed no significant difference in satisfaction with scenario-based simulation teaching, SPOCs, or the interactive educational software program Zuvio, while the mean and standard deviation in fourth year students were higher than fifth year students. This result was likely caused by differences in group background and because fourth-year students were preparing for their internship and thus responded more positively to innovative teaching. The fifth-year students had already completed 1 year of clinical internship and were preparing for national entrance examinations; therefore, they were more committed to preparing for mock examinations in school than they were committed to studying for elective courses.

**Table 5:** Independent sample *t*-test results for student satisfaction with innovative teaching.

**Variables**	**Fourth-year students (*****n*** **=** **61)**		**Fifth-year students (*****n*** **=** **65)**		***t*-value**	**Degree of freedom**	**Significance (two-tailed)**
	**M**	**SD**	**M**	**SD**			
Scenario-based simulation teaching	4.667	0.385	4.468	0.712	1.926	122	0.056
SPOCs	4.667	0.460	4.459	0.723	1.909	122	0.059
Interactive educational software Zuvio	4.652	0.476	4.425	0.768	1.972	122	0.051

## Discussion and Conclusion

### Measurement Model Analysis

The results of this study exhibited that after participating in innovative teaching, the fourth- and fifth-year students exhibited significant differences in overall revised OSCE scores; accordingly, internship experience and other factors contributed to higher overall revised OSCE scores. Subsequently, the differences in scores between the two groups in the three OSCE dimensions were analyzed; significant differences were observed in the BLS and SDH scores. The mean BLS score of the fifth-year students was 9.59 points higher than that of the fourth-year students, and the two groups exhibited a considerable 22.05-point difference in SDH scores. Accordingly, internship experience and other factors related to learning experience contributed to higher scores on the three OSCE dimensions. According to the internal discussion of the course development group, SDH was the most difficult to learn, BLS required basic knowledge and skills, and AMI was of medium level of difficulty. The varying difficulty levels across OSCE might have caused the two groups to exhibit non-significant differences in the AMI dimension.

After participating in innovative teaching, the fourth- and fifth-year students exhibited significant differences in AMI SA1; BLS SA1, SA2, and SA3; and SDH SA3. Moreover, the fifth-year students had higher scores than the fourth-year students. Specifically, the two groups exhibited a considerable 21.29-point difference in SDH SA3. The high difficulty level of SDH might have caused a marked difference in scores between the two groups. Accordingly, internship experience and factors related to learning experience (e.g., higher seniority) greatly influenced the SA3 score for SDH, the most difficult subject. Bambini (15) proposed that the complexity of the simulation should be built according to the learner's level and match their competency. To construct the scenario lesson plans, which approximate clinical practices and complexity, the lesson planner should consider students' competency and complexity of materials simultaneously. Furthermore, the level of difficulty in the curriculum is related to the revised OSCE checklist, as seen in the significant difference between fourth and fifth year students in the total OSCE score and in SA 3. Recommendations for future development of the OSCE checklist based on simulation curricula include formulating program year- and subject-appropriate test items paired to the level of difficulty with SA 1, SA 2 and SA 3 in order to increase student-centered KSA.

In the satisfaction survey, fourth-year students had significantly higher scores than fifth-year students on three OSCE dimensions and on overall satisfaction, which may be considered the overall evaluation of the proposed innovative teaching method. The different learning plans students had may have affected the evaluation results. The fourth-year students, who were preparing for their internships, exhibited high satisfaction with innovative teaching, especially in scenario-based simulation.

Fourth-year students had an overall satisfaction rate of 96.6% for SPOCs, which was higher than that of fifth-year students by 7.2%. The different learning plans students had may have resulted in the considerable difference between the two groups in terms of SPOC satisfaction rate. Specifically, fourth-year students were preparing for their internships, whereas fifth-year students were preparing for exams. Different learning goals between the two groups might have affected their satisfaction levels. Another possible factor influencing satisfaction is whether the SPOC recordings were attractive to the students. The overall optimization of online courses in the future may yield superior results. In terms of platform operation, both groups found Ewant easy to use and helpful for reviewing physical assessments, basic medicine, and medical–surgical nursing; both groups of students were highly satisfied with the application of SPOCs to autonomous learning and the use of the flipped classroom approach for course preview and review. Both the fourth- and fifth-year students had satisfaction rates of 98 and 89.5% toward Zuvio, respectively, and such high satisfaction may be attributable to the software's diverse functions. The fourth-year students had more experience using Zuvio, whereas the fifth-year students—who had just returned to school after a year-long internship—had little experience using it in class, despite its simple user interface. Whether usage frequency affected satisfaction requires further discussion.

Because of the increase in confirmed COVID-19 cases, numerous schools stopped offering onsite education and switched to remote learning (10). Previous literature has already reported the advantages of replacing MOOCs with SPOCs because there were some challenges of MOOCs (9, 16–18). The findings of this study demonstrated that SPOCs support the effectiveness of small-scale blended learning, and that this learning mechanism enhances the comprehensive and in-depth learning experiences for students while providing instructors with flexible and feasible teaching models simultaneously. Such model will provide a convenient way for instructors to understand students' learning needs and behaviors through learning hours, achievement rates, and formative (on-going feedback between students and instructors) and summative (tallying of test scores) evaluations. However, this study is an action research through a practicum course and we could not utilize only one mode of instruction while withholding others to protect the students' rights and interests. Future studies to compare between different modes of instructions such as ones in this study may be conducted in elective or non-core courses. Another limitation of the present study was that the students in the experimental group cannot prevent from Hawthorne effect since the research process cannot adopt a double-blind design in practical.

## Data Availability Statement

The original contributions presented in the study are included in the article/supplementary material, further inquiries can be directed to the corresponding author/s.

## Ethics Statement

The studies involving human participants were reviewed and approved by St. Martin De Porres Hospital (IRB No.: 18B-012 and Date of Approval: 2019/11/1). The patients/participants provided their written informed consent to participate in this study.

## Author Contributions

Y-KO and L-PT: study conception and design. L-PT: data collection. L-PT and L-PH: data analysis and interpretation. Y-KO, L-PT, and T-HH: drafting of the article and critical revision of the article. All authors have read and agreed to the published version of the manuscript.

## Funding

This study was funded by the St. Martin De Porres Hospital (HR54-P1903) and the Allied Advanced Intelligent Biomedical Research Center (A21BRC) under the Higher Education Sprout Project of Ministry of Education.

## Conflict of Interest

The authors declare that the research was conducted in the absence of any commercial or financial relationships that could be construed as a potential conflict of interest.

## Publisher's Note

All claims expressed in this article are solely those of the authors and do not necessarily represent those of their affiliated organizations, or those of the publisher, the editors and the reviewers. Any product that may be evaluated in this article, or claim that may be made by its manufacturer, is not guaranteed or endorsed by the publisher.
